# Acupuncture and Depth: Future Direction for Acupuncture Research

**DOI:** 10.1155/2014/871217

**Published:** 2014-07-08

**Authors:** You Li Goh, Chin Ee Ho, Baixiao Zhao

**Affiliations:** ^1^School of Acupuncture-Moxibustion and Tuina, Beijing University of Chinese Medicine, 11 North East Third Ring Road, Chaoyang District, Beijing 100029, China; ^2^School of Biological Sciences, College of Science, Nanyang Technological University, Singapore 639798; ^3^Dongfang Hospital, Second Affiliated Hospital of Beijing University of Chinese Medicine, Beijing 100078, China

## Abstract

The research on acupuncture has increased steadily over the years and regular review and revision of the direction of future acupuncture research are necessary. This paper aims to review and explore the significance of acupuncture depth in modern acupuncture research. Searches conducted in Science Direct and China National Knowledge Infrastructure (CNKI) databases reflected a lack of focus on depth of acupuncture. We propose that the research trends of acupuncture should progress to the depth of insertion. It is suggested that future acupuncture research, especially randomized controlled trials (RCTs), should take into consideration the depth of insertion. Comparison between databases using different language of medium suggests the need for international collaboration of researchers from the same field. It is also crucial to inherit and innovate traditional medicine (TM) through modern technology. The use of bibliometric method is also suitable for development of TM research trends. Acupuncture and depth should be considered as one of the future directions of acupuncture research.

## 1. Introduction

The research of traditional medicine (TM) and acupuncture has increased steadily in the recent years. Globalization and the World Wide Web have allowed researchers from different countries to obtain the latest development of all research studies. It is appropriate to review and analyze the trends of the research focus methodology by all researchers in the same fie.

Acupuncture depth is an area of study left unexplored by many researchers. Deep or superficial insertion of needle through the skin would directly affect the type and amount of tissues that it excites. Numerous randomized controlled trials (RCTs) have been conducted to test the efficacy of acupuncture and the presence of placebo effect. On the contrary, relatively less emphasis has been placed on acupuncture depth. Researchers using functional magnetic resonance (fMRI) to test mild cognitive impairment found that deep muscle insertion of acupuncture is necessary to achieve appreciable clinical effects [1–3]. Others have used ultrasound scans to guide the specific depth for achieving acupuncture sensation [4]. Such findings correspond to the Layer Analysis as described in the Yellow Emperor's Inner Classic [5]. Hence, we hypothesize that there could be a relationship between the efficacy of acupuncture treatment and the depth of insertion.

We believe that acupuncture depth should be considered an area of research in the future. The use of bibliometric indicators is gaining popularity to evaluate clinical research [6]. In order to determine the consideration of depth in recent acupuncture researches, this review also explores the bibliometric data of acupuncture depth.

## 2. Lack of Acupuncture Depth Research

A search in databases Science Direct and China National Knowledge Infrastructure (CNKI) using the follow strategies was conducted. For Science Direct, “acupuncture” or “moxibustion” or “acupuncture-moxibustion” or “needling” and “depth” were used. For search in CNKI, “acupuncture” (*针刺*) or “moxibustion” (*灸*) or “acupuncture-moxibustion” (*针灸*) and “depth” (*深度*) or “layer” (*层次*) (not “Layer Analysis method” (*层次* *分* *析* *法*)) were used. Similar search strategies were employed for publications on efficacy using the terms “efficacy,” “*效应*,” or “*有效率*”. Only articles from the recent ten years (2004–2013) were included. All languages available were included. Microsoft Excel was used to collect the data for analysis as described by previous studies [7].

**Figure 1:**
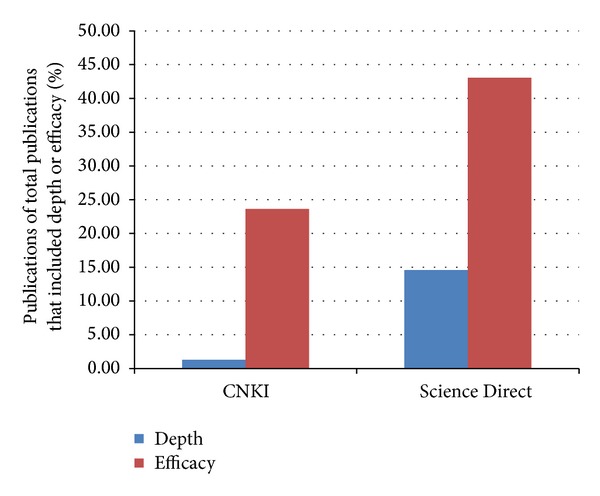
Percentage of total publications in CNKI and Science Direct on acupuncture, acupuncture-moxibustion, moxibustion, and needling from 2004 to 2013 that included depth (blue) or efficacy (red).

The search yielded a total of 16517 and 79786 publications in Science Direct and CNKI, respectively. The search also found that 14.58% of publications in Science Direct and 1.32% in CNKI on acupuncture are depth related, while that of clinical efficacy research is 43.06% and 23.65%, respectively (Figure 1). This highlights the lack of study in the acupuncture depth arena, warranting more focus on acupuncture and depth research.

## 3. Proposed Progression of Research Trends

**Figure 2:**
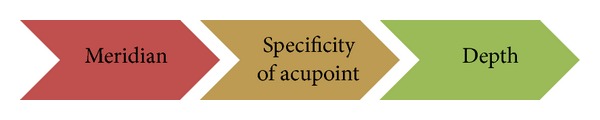
A proposed possible progression of research trends of acupuncture.

The research on TM has reached a point whereby researchers should pause and reflect on its current directions. The research on acupuncture started off focusing on the meridian effects [8], which was one of the essential building blocks of traditional Chinese medicine (TCM) theory and acupuncture. In the recent years, specificity of meridian acupoint has also become a research hotspot [9–13]. As we progress from meridian studies to specificity of a meridian acupoint, we propose more research to study the variability of effects caused by different insertion depth on a selected point (Figure 2). Such a progression of research trends is in line with the development of modern technology such as ultrasound, fMRI, and laser, which were not available in the past.

## 4. Depth of Insertion

The depth of insertion, invasion of needles, and other techniques are always varying among different practitioners. Acupuncture textbooks stating recommended ranges for the depth of insertion are mainly for safety purpose [14]. This review emphasizes the importance of depth of insertion and puts forward the consideration of depth in future acupuncture research.

### 4.1. Factor for Guideline of Acupuncture Control Procedure

Numerous acupuncture researches had focused on the placebo effect of acupuncture [15–18] and used minimal or sham acupuncture as control to real acupuncture [19–23]. Studies had questioned the accuracy of the definition of placebo [24]. Others also questioned whether minimal, superficial, or sham acupuncture procedures are acceptable as inert placebo controls [25, 26]. Another review concluded that clear guideline to assess acupuncture control procedures will improve the quality of RCTs and systematic reviews [27]. Such studies are indicators that the research on depth of acupuncture is crucial for acupuncture research since the common discrepancy is the lack of understanding on effect of depth of insertion on efficacy of acupuncture. It is also evident that acupuncture control procedures guideline should consider the depth of real and control acupuncture.

### 4.2. Modern Development of Acupuncture

Developments of acupuncture have evolved from the traditional methods and depth of insertion is crucial in some, such as Fu's subcutaneous needling (FSN). FSN is a modern innovative style of acupuncture which focused on the subcutaneous depth [28, 29]. Studies have been done on FSN, such as clinical trial of FSN on low back pain which suggested its immediate and safe effects [30]. Such innovation suggests the clinical effect of acupuncture apart from the traditional insertion and signifies the importance of depth in modern acupuncture research.

### 4.3. An Example of Inheriting and Innovating TM through Modern Technology

Effective clinical effects produced by renowned acupuncturists are significantly related to the depth they needle for different diseases or point. It is crucial to inherit and explore these experiences by research in the fields of acupuncture depth. We believe that the key to developing TM is to inherit the traditional methods and innovate with the modern technology in order to achieve better healthcare for mankind.

Depth of insertion could only be estimated by acupuncturists in the past due to limitations of expertise. However, with modern technological advancements, such as laser technology, the depth of stimulation can be examined. There are researchers who have investigated specifically on the violet laser acupuncture (405 nm) which has a penetration depth of 1-2 mm [31–35]. The same team also carried out investigations to compare differences between violet laser acupuncture and red laser acupuncture (685 nm; 3-4 mm) and found different effects on heart rate variability [36]. Such findings showing two specific depths of penetration producing different effects are a breakthrough on research on TM. Clinical trials were also conducted to test the effectiveness and efficacy of laser acupuncture [37–39]. Research on acupuncture should be in tandem with modern technology. In addition to innovation, one has to keep in mind the importance of inheritance. In this case, the principle of “point location according to proportional bone measurement” [40] (*骨度分寸*) has to be abided. The ratio or proportion of the depth of insertion relative to the part of the body being needled should be considered. This is a display of how we can both inherit and innovate TM for its development.

It is crucial to inherit from experienced mentors in order to find a focus for the research. The authors' mentor, Professor Yang Jia-san [41], had always emphasized the depth of insertion of the needle and hence the authors hope to inherit from and innovate his experiences. TM places immense emphasis on the inheritance of experiences from mentors and such experiences are deemed important treasures of TM. Such experiences left behind by mentors and practitioners before us are crucial for the inheritance and innovation of TM.

### 4.4. Proposal to Determine the Precise Point of Insertion

**Figure 3:**
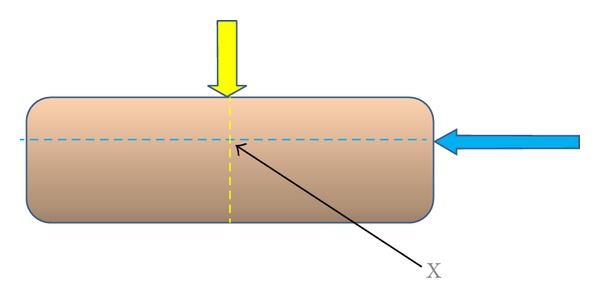
A simulated diagram of acupoint chosen guided by the traditional meridian and collateral theory which forms the horizontal axis (yellow), while depth of chosen acupoint would form the vertical axis (blue) of Point X. Point X is the proposed precise point of insertion.

Through the abovementioned method, we can propose determining which tissues or levels would create optimal treatment effect. Taking the acupuncture point on the meridian and collateral as the horizontal axis of the point of insertion, the precise acupoint, X, can be determined by finding the depth of insertion, which serves as the vertical axis (Figure 3). The meridian and collateral theory would be the guide for choice of acupoint on the horizontal axis, while more research is warranted to study the depth of the chosen acupoint.

## 5. Research Methodology 

The amount of research done on alternative, complementary, and traditional medicine has increased steadily in the recent years. It is appropriate to review and analyze the trends of the research methodology by all researchers in the same field. This review serves as an example of comparing the research from different parts of the world through two databases which use two different main languages of medium.

### 5.1. Comparison between CNKI and Science Direct

**Figure 4:**
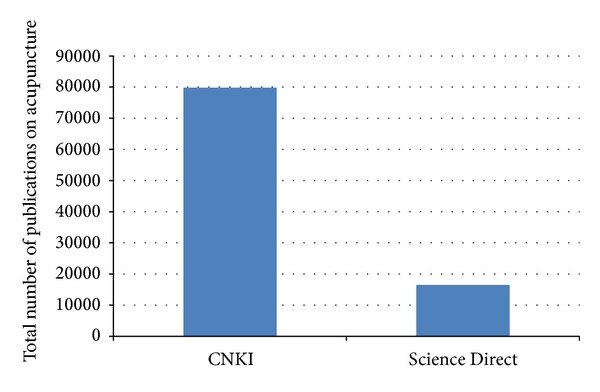
The total publications on acupuncture, acupuncture-moxibustion, moxibustion, and needling that were indexed in Science Direct and CNKI from 2004 to 2013.

**Figure 5:**
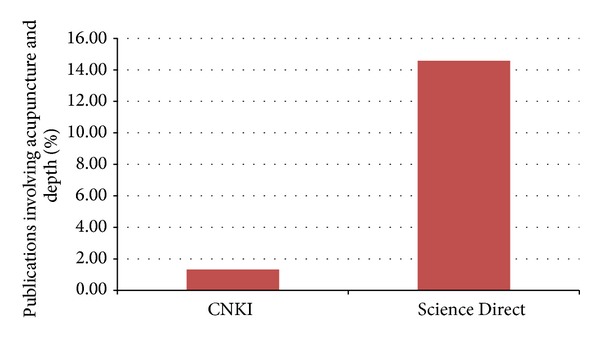
Percentage of total publications in CNKI and Science Direct on acupuncture, acupuncture-moxibustion, moxibustion, and needling from 2004 to 2013 that included depth.

Comparison between CNKI and Science Direct provides researchers with an apt opportunity to pause and reflect on the trend of future direction of research. Comparison between databases from different countries could be worthwhile. The number of acupuncture-related publications in CNKI is about five times that in Science Direct (Figure 4). Interestingly, the percentage of research in Science Direct done on depth was more than ten times that of CNKI (Figure 5). 1.32% of CNKI reports involved depth while Science Direct had 14.58%. This result is inversed as compared to the total publications on acupuncture from both databases. This suggests the difference in focus of research from both databases, with respect to depth of acupuncture.

Paradoxically, although acupuncture and the Layer Analysis method [5] originate from China, the percentage of research indexed in the area of acupuncture depth in Science Direct was ten times as compared to CNKI, the biggest published research database in China (Figure 5). In addition, the search from both databases also points to the lack of research focus on depth generally. The authors believe that the amount of research that considers depth in both CNKI and Science Direct should be and would be increased in the future.

This result indicates that studies on acupuncture from the two databases had relatively different focus. Such comparisons are crucial for researchers to determine the focus of their research. It also indicates that international collaborations are warranted for research in acupuncture to build dynamic and innovative research.

Future studies are necessary to compare and analyze the directions of research teams from different parts of the world, in order to work hand in hand to propel the development of TM. This review shows that such comparison between different databases could produce worthwhile results.

### 5.2. Bibliometric Method

Bibliometric studies are gaining popularity since the amount of research done increases every year. Bibliometric analysis is an important tool for TM researchers in order to consider the trend of research of TM. More bibliometric studies are warranted in the field of TM in order to determine the future trends. Based on the holism concept of TCM, researchers are familiar with the importance of consideration of the big picture. Hence, this paper proposed the importance of bibliometric data and studies on future TM research.

This paper aims to consider all kinds of research in order to obtain information from all possible research teams. Future studies are warranted to consider more information in order to propel more detailed research in this field.

## 6. Conclusion

The plentiful variables that exist in acupuncture research are the reason for the lack of scrutiny on the depth of acupuncture. However, as research progresses, one should also focus on the amount of tissue invaded and types of tissues excited. Along with the advancements in imaging, laser technologies, and so forth, research on acupuncture depth could also progress. We suggest that future acupuncture research, especially RCTs, should take into consideration the depth of insertion. In addition, the use of bibliometric method is crucial for future development of TM research trends too.

## 7. Future Direction

Acupuncture depth could possibly be an important aspect in future research arena of traditional medicine (TM). The use of bibliometric indicators to analyze and compare the existing research from all parts of the world is warranted too.
